# Generalized Finite-Length Fibonacci Sequences in Healthy and Pathological Human Walking: Comprehensively Assessing Recursivity, Asymmetry, Consistency, Self-Similarity, and Variability of Gaits

**DOI:** 10.3389/fnhum.2021.649533

**Published:** 2021-08-09

**Authors:** Cristiano Maria Verrelli, Marco Iosa, Paolo Roselli, Antonio Pisani, Franco Giannini, Giovanni Saggio

**Affiliations:** ^1^Department of Electronic Engineering, University of Rome Tor Vergata, Rome, Italy; ^2^Department of Psychology, Sapienza University of Rome, Rome, Italy; ^3^Laboratory for the Study of Mind and Action in Rehabilitation Technologies, Istituto di Ricovero e Cura a Carattere Scientifico Santa Lucia Foundation, Rome, Italy; ^4^Department of Mathematics of University of Rome Tor Vergata, Rome, Italy; ^5^Institut de Recherche en Mathématique et Physique, Universite' Catholique de Louvain, Ottignies-Louvain-la-Neuve, Belgium; ^6^Department of Brain and Behavioral Sciences, University of Pavia, Pavia, Italy; ^7^Istituto di Ricovero e Cura a Carattere Scientific Mondino Foundation, Pavia, Italy

**Keywords:** gait analysis, walking gait, asymmetry, self-similarity, golden ratio, fibonacci sequence, locomotion, neuroscience

## Abstract

Healthy and pathological human walking are here interpreted, from a temporal point of view, by means of dynamics-on-graph concepts and generalized finite-length Fibonacci sequences. Such sequences, in their most general definition, concern two sets of eight specific time intervals for the newly defined *composite gait cycle*, which involves two specific couples of overlapping (left and right) gait cycles. The role of the golden ratio, whose occurrence has been experimentally found in the recent literature, is accordingly characterized, without resorting to complex tools from linear algebra. Gait recursivity, self-similarity, and asymmetry (including double support sub-phase consistency) are comprehensively captured. A new gait index, named Φ*-bonacci gait number*, and a new related experimental conjecture—concerning the position of the foot relative to the tibia—are concurrently proposed. Experimental results on healthy or pathological gaits support the theoretical derivations.

## 1. Introduction

Four time intervals—associated with the durations of gait cycle, swing, stance and double support phases—characterize, from a temporal point of view, symmetric and recursive human walking (Dugan and Bat, 2005). Recently, the ratio between swing and double support phases durations has been experimentally recognized in Iosa et al. (2013)^1^ to be close, in healthy subjects symmetrically and recursively walking at comfortable speed of about 4 km/h (Cavagna and Margaria, 1966), to the golden ratio $\phi =\left(1+\sqrt{5}\right)/2\approx 1.618$. Such an irrational number ϕ is the positive solution to the equation *x*^2^ = 1 + *x*. It is related to the Euclid's problem of cutting in a self-proportional way a given straight segment (Iosa et al., 2017, 2019). In this light, ϕ turns out to describe self-similarity in symmetric walking (Iosa et al., 2019). Indeed, most of the literature agrees that the foot off reliably occurs at 60–62% of a physiological gait when the subject is (symmetrically and recursively) walking at comfortable speed^2^. On the other hand, it has been also experimentally shown that patients with Parkinson's Disease—known to be characterized by tremor at rest, rigidity, akinesia, or bradykinesia, and postural instability—have such a smooth, graceful and melodic flow of movement being reduced, with their gait self-similarity being altered (Iosa et al., 2016b). Notice how all the experimental evidences above move along the direction of using temporal gait analyses to complement, in clinical or general performance evaluations (Salarian et al., 2004; Wang et al., 2012; do Carmo Vilas-Boas and Cunha, 2016; Ren et al., 2016; Serrao et al., 2017; Ricci et al., 2019b), the classical gait analyses including motion analysis, dynamic electromyography, force plate recordings, energy cost measurements or energetics, measurement of stride characteristics (Dugan and Bat, 2005; Greene et al., 2010; Saggio and Sbernini, 2011). However, human walking naturally includes asymmetric and non-recursive components, especially in pathological cases, so that at least eight (in place of four) time intervals have to be considered. These time intervals include the gait cycle, swing, stance, double support durations for both the left and right lower limbs (Marino et al., 2020).

**Figure 1:**
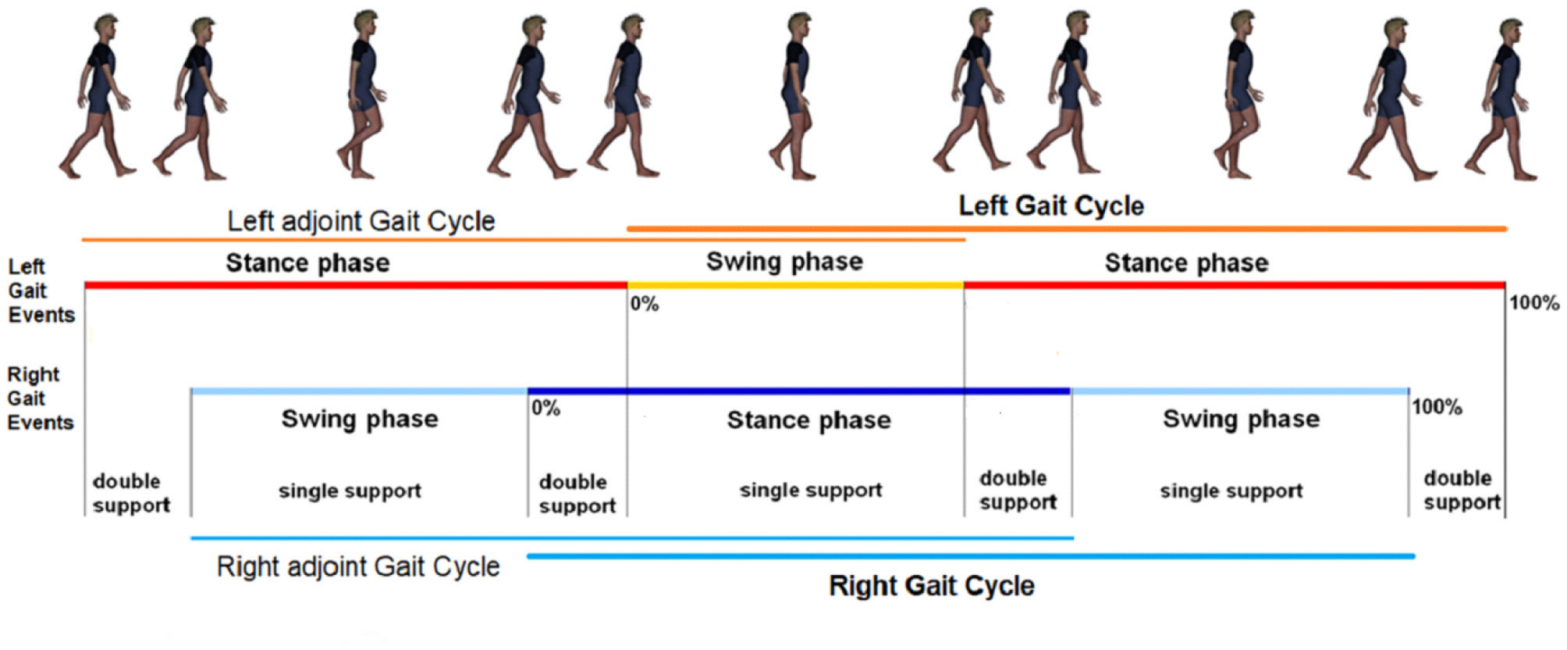
Partitions of the walking gaits.

This work definitely exploits the ideas underlying a fractal approach to the question^3^, in which the larger scale structure resembles the subunit structure. It moves along the direction of providing special interest to the simplest and most general way of transformation when a new domain is composed of two previous ones, with a consequent internal evolutionary process including the generation of a self-referential loop^4^. In particular, this paper provides original mathematically-founded arguments addressing the ¬-{symmetry and recursivity} question above. As in Marino et al. (2020), the crucial role of ϕ is found to be intrinsically related to the mathematical description of the human walking, rather than to be associated with the special solution constituted by a temporally self-similar gait^5^. However, differently from Marino et al. (2020), no complex tools from linear algebra, associating special ϕ-dependent subspaces with a common temporal model for human walking and running gaits, are here employed. Instead, human walking is here described in terms of generalized finite-length Fibonacci sequences (Horadam, 1961)^6^ and dynamics-on-graph concepts (an interpretation in terms of Shannon entropy is also presented in Appendix A). Furthermore, in contrast to Marino et al. (2020), the new mathematical concept of *composite gait cycle* is here innovatively analyzed: it involves (see Figure 1) two specific couples of overlapping gait cycles, namely the left and right gait cycles and the *adjoint* right and left gait cycles, while extending the idea of stride-to-stride interval (Kavanagh et al., 2006) and step-by-step interval (Potdevin et al., 2007). The analysis presented in this paper generalizes the one in Iosa et al. (2013), as much as the new index of section 2, named Φ*-bonacci gait number*, constitutes the most straightforward generalization of the gait ratio in Iosa et al. (2013) to the case in which non-{symmetric and recursive} components of walking (including the concept of double support consistency) occur. Furthermore, differently from the area of the *Synchronicity Rectangle* in Marino et al. (2020), such a new index takes its minimum zero-value just when the *enforced adjoint symmetric self-similarity* occurs. The above index, which can be naturally extended to even assess *gait index variability* along past walking gaits (Appendix B), also innovatively involves a term relying on a new experimental conjecture (section 2) that opens new analysis and diagnosis perspectives on the internal analysis of the double support phase. An experimental support to the results of this paper is finally provided in section 3, with a detailed discussion being reported in section 4.

## 2. Materials and Methods

### 2.1. Walking Phase Partition

**Figure 2:**
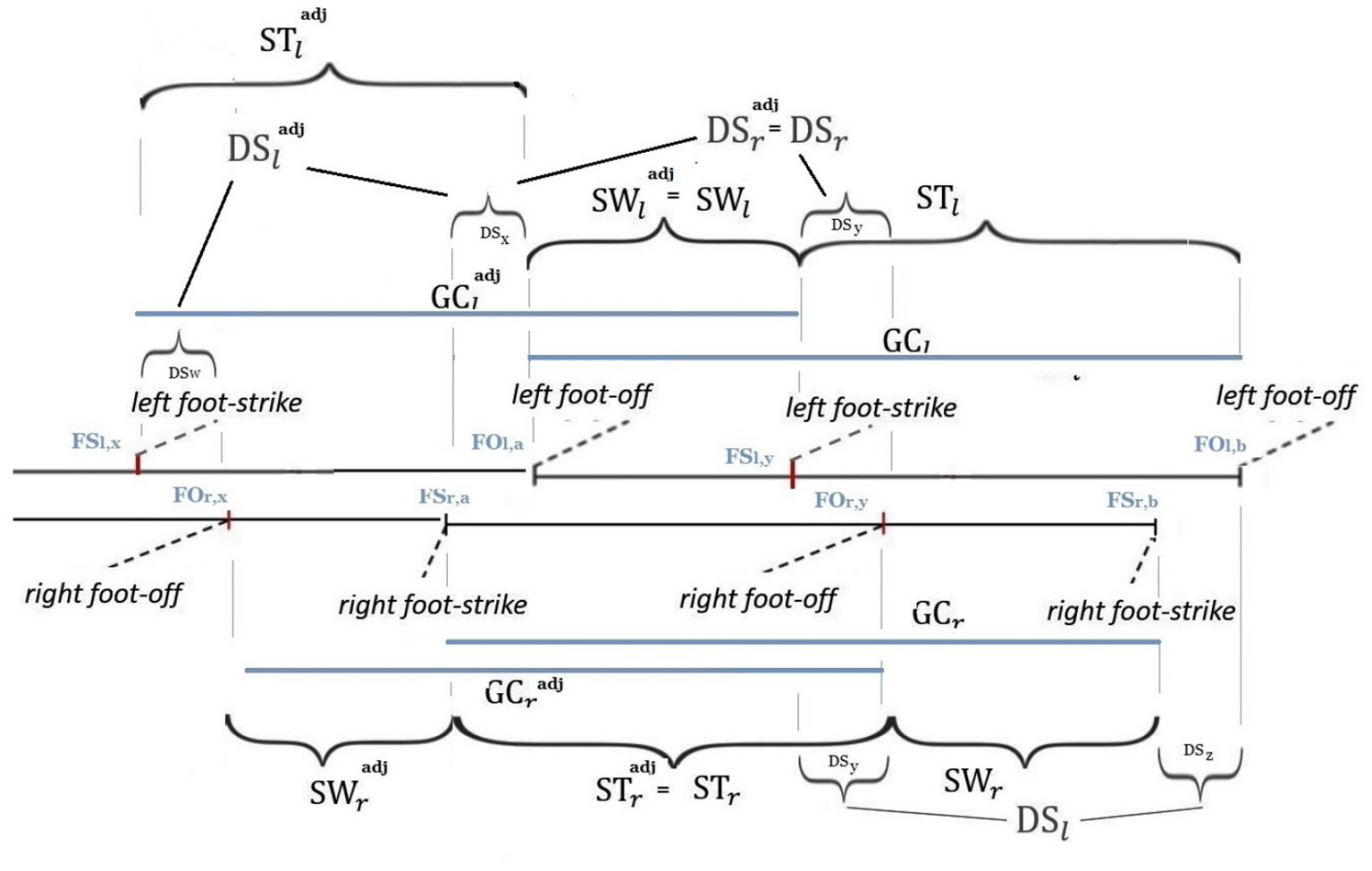
*Composite gait cycle*: right and left gait cycles and *adjoint* right and left gait cycles.

Walking is defined as the bipedal locomotion gait (Kirtley, 2006; Iosa et al., 2013) such that: (1) at least one limb is in contact with the ground; (2) the contact phases are alternated by the two limbs. In particular, condition 1. excludes, in walking, the following possible state of the limbs: {no limbs in contact with the ground}. Even though each gait cycle conventionally starts and finish with consecutive foot strikes of the same foot (being formed by the stance and the swing of the same limb), we here adopt, as in Marino et al. (2020), the compactly comprehensive modeling of Figure 2, which defines the right and left gait cycles, with duration GC_*r*_, GC_*l*_, as the time intervals (or phases) between two consecutive strikes of the right foot, namely FS_*r,a*_ and FS_*r,b*_, and two consecutive lift off of the left foot, namely FO_*l,a*_ and FO_*l,b*_, respectively. This way, as we shall see, the same right swing phase appearing in the left stance phase is the one that is involved in the definition of the right gait cycle (see Figure 2). Analogously define the *adjoint* right and left gait cycles, with duration ${\text{GC}}_{r}^{\text{adj}}$, ${\text{GC}}_{l}^{\text{adj}}$, as the time intervals (or phases) between two consecutive lift off of the right foot, namely FO_*r,x*_ and FO_*r,y*_, and two consecutive strikes of the left foot, namely FS_*l,x*_ and FS_*l,y*_, respectively, with FO_*r,x*_ and FS_*l,x*_ immediately preceding FS_*r,a*_ and FO_*r,x*_, respectively (see Figure 2). Here: GC stands for Gait Cycle; FS stands for Foot Strike; FO stands for Foot Off; *r* and *l* stand for right and left, respectively; adj stands for adjoint. Thereafter: ST stands for STance; SW stands for SWing; DS stands for Double Support.

**Remark:** As shown in Figure 2, each stance contains the contralateral swing besides the double support sub-phase with the left foot behind and the right foot ahead and the double support sub-phase with the right foot behind and left foot ahead. This will be crucial in defining the double support consistency concept of the reminder of this paper. On the other hand, the need of considering the adjoint gait relies on the fact that the assumption of gait symmetry and recursivity often decays in pathological walking.

First refer to the right and left gait cycles, with duration GC_*r*_, GC_*l*_. The phase in the right (resp., left) gait cycle in which the right (resp., left) limb is in-contact with the ground is named right (resp., left) stance and has time duration ST_*r*_ (resp., ST_*l*_). The right swing and left swing phases are defined as the time intervals in which the right (resp., left) limb is not in contact with the ground during the right (resp., left) gait cycle (Iosa et al., 2013). Their durations are given by^7^:

(1) $$\begin{array}{l} {\text{SW}}_{r}={\text{GC}}_{r}-{\text{ST}}_{r},\;{\text{SW}}_{l}={\text{GC}}_{l}-{\text{ST}}_{l}. \end{array}$$

As aforementioned, during walking, there cannot be a double float phase in which both feet are off the ground (condition 1.), so that the left swing phase (resp., right swing phase) must be entirely contained in the right stance phase (resp., left stance phase). The non-negative difference between their durations leads to the definition of the right (resp., left) double support phase that is entirely contained in the right gait cycle (resp., left gait cycle). Its duration DS_*r*_ (resp., DS_*l*_) is given by:

(2) $$\begin{array}{l} {\text{DS}}_{r}={\text{ST}}_{r}-{\text{SW}}_{l}\;\left({\text{DS}}_{l}={\text{ST}}_{l}-{\text{SW}}_{r}\right). \end{array}$$

The durations DS_*r*_ and DS_*l*_ in turn satisfy DS_*r*_ = DS_*x*_ + DS_*y*_, DS_*l*_ = DS_*y*_ + DS_*z*_, where DS_*x*_, DS_*y*_, DS_*z*_ denote the durations of the double support sub-phases highlighted in Figure 2. On the other hand, the same quantities can be introduced for the *adjoint* right and left gait cycles, with duration ${\text{GC}}_{r}^{\text{adj}}$, ${\text{GC}}_{l}^{\text{adj}}$. They are denoted by ${\text{ST}}_{r}^{\text{adj}}$, ${\text{ST}}_{l}^{\text{adj}}$, ${\text{SW}}_{r}^{\text{adj}}$, ${\text{SW}}_{l}^{\text{adj}}$, ${\text{DS}}_{r}^{\text{adj}}$, ${\text{DS}}_{l}^{\text{adj}}$ and satisfy by definition (see Figure 2): ${\text{DS}}_{r}^{\text{adj}}={\text{DS}}_{r}$, ${\text{SW}}_{l}^{\text{adj}}={\text{SW}}_{l}$, ${\text{ST}}_{r}^{\text{adj}}={\text{ST}}_{r}$. Again, the durations ${\text{DS}}_{r}^{\text{adj}}$ and ${\text{DS}}_{l}^{\text{adj}}$ in turn satisfy ${\text{DS}}_{r}^{\text{adj}}={\text{DS}}_{x}+{\text{DS}}_{y}$, ${\text{DS}}_{l}^{\text{adj}}={\text{DS}}_{w}+{\text{DS}}_{x}$, where DS_*x*_ denotes the duration of the double support sub-phase highlighted in Figure 2.

The following proposition holds. It generalizes the corresponding one in Marino et al. (2020), while it allows to extend the analysis of Iosa et al. (2013) to pathological gaits in which the inequalities: GC_*r*_ ≠ GC_*l*_ (resp., ${\text{GC}}_{r}^{\text{adj}}\neq {\text{GC}}_{l}^{\text{adj}}$), ST_*r*_ ≠ ST_*l*_ (resp., ${\text{ST}}_{r}^{\text{adj}}\neq {\text{ST}}_{l}^{\text{adj}}$), SW_*r*_ ≠ SW_*l*_ (resp., ${\text{SW}}_{r}^{\text{adj}}\neq {\text{SW}}_{l}^{\text{adj}}$), DS_*r*_ ≠ DS_*l*_ (resp., ${\text{DS}}_{r}^{\text{adj}}\neq {\text{DS}}_{l}^{\text{adj}}$) possibly occur.

**Proposition 1:***Given the 16 time intervals durations* GC_*r*_, GC_*l*_, ST_*r*_, ST_*l*_, SW_*r*_, SW_*l*_, DS_*r*_, DS_*l*_, ${\text{GC}}_{r}^{\text{adj}}$, ${\text{GC}}_{l}^{\text{adj}}$, ${\text{ST}}_{r}^{\text{adj}}$, ${\text{ST}}_{l}^{\text{adj}}$, ${\text{SW}}_{r}^{\text{adj}}$, ${\text{SW}}_{l}^{\text{adj}}$, ${\text{DS}}_{r}^{\text{adj}}$, ${\text{DS}}_{l}^{\text{adj}}$ *(under* ${\text{DS}}_{r}^{\text{adj}}={\text{DS}}_{r}$, ${\text{SW}}_{l}^{\text{adj}}={\text{SW}}_{l}$, ${\text{ST}}_{r}^{\text{adj}}={\text{ST}}_{r}$*), they define a composite walking cycle if the following eight equality constraints are satisfied:*

(3) $$\begin{array}{l} \;{\text{ST}}_{r}={\text{DS}}_{r}+{\text{SW}}_{l},\;{\text{GC}}_{r}={\text{DS}}_{r}+{\text{SW}}_{l}+{\text{SW}}_{r}\\ \;{\text{ST}}_{l}={\text{DS}}_{l}+{\text{SW}}_{r},\;{\text{GC}}_{l}={\text{DS}}_{l}+{\text{SW}}_{r}+{\text{SW}}_{l}.\\ {\text{ST}}_{r}^{\text{adj}}={\text{DS}}_{r}^{\text{adj}}+{\text{SW}}_{l}^{\text{adj}},\;{\text{GC}}_{r}^{\text{adj}}={\text{DS}}_{r}^{\text{adj}}+{\text{SW}}_{l}^{\text{adj}}+{\text{SW}}_{r}^{\text{adj}}\\ {\text{ST}}_{l}^{\text{adj}}={\text{DS}}_{l}^{\text{adj}}+{\text{SW}}_{r}^{\text{adj}},\;{\text{GC}}_{l}^{\text{adj}}={\text{DS}}_{l}^{\text{adj}}+{\text{SW}}_{r}^{\text{adj}}+{\text{SW}}_{l}^{\text{adj}}. \end{array}$$

### 2.2. Generalized Finite-Length Fibonacci Sequences and Classification

Define the two (right and left) chains that are represented by the sequences:

$$\begin{array}{l} {\text{DS}}_{r}\;\to \;{\text{SW}}_{l}\;\to \;{\text{ST}}_{r}\;\to \;{\text{GC}}_{r}\\ {\text{DS}}_{l}\;\to \;{\text{SW}}_{r}\;\to \;{\text{ST}}_{l}\;\to \;{\text{GC}}_{l}, \end{array}$$

along with their *adjoint* versions^8^:

$$\begin{array}{l} {\text{DS}}_{r}^{\text{adj}}\;\to \;{\text{SW}}_{l}^{\text{adj}}\;\to \;{\text{ST}}_{r}^{\text{adj}}\;\to \;{\text{GC}}_{r}^{\text{adj}}\\ {\text{DS}}_{l}^{\text{adj}}\;\to \;{\text{SW}}_{r}^{\text{adj}}\;\to \;{\text{ST}}_{l}^{\text{adj}}\;\to \;{\text{GC}}_{l}^{\text{adj}}. \end{array}$$

The symmetric and recursive case and the (more general) non-{symmetric and recursive} one will be distinguished, in order to make the asymmetric walking be viewed as a natural extension of the symmetric and recursive one.

*Symmetric and recursive walking*. The following equalities: ${\text{GC}}_{r}={\text{GC}}_{l}={\text{GC}}_{r}^{\text{adj}}={\text{GC}}_{l}^{\text{adj}}=\text{GC}$, ${\text{ST}}_{r}={\text{ST}}_{l}={\text{ST}}_{l}^{\text{adj}}={\text{ST}}_{r}^{\text{adj}}=\text{ST}$, ${\text{SW}}_{r}={\text{SW}}_{l}={\text{SW}}_{r}^{\text{adj}}={\text{SW}}_{l}^{\text{adj}}=\text{SW}$, ${\text{DS}}_{r}={\text{DS}}_{l}={\text{DS}}_{l}^{\text{adj}}={\text{DS}}_{r}^{\text{adj}}=\text{DS}$ hold in symmetric and recursive walking, so that the two above chains and their *adjoint* versions collaps into one, namely into DS → SW → ST → GC.

The following proposition holds, whose proof directly comes from (3), once it is specialized to the symmetric and recursive case.

**Proposition 2:***The chain DS* → *SW* → *ST* → *GC represents a (generalized)* (*a, b*)*-generated 4-length Fibonacci sequence*^9^ *of the form:*

(4) $$\begin{array}{l} a,\;b,\;c,\;d \end{array}$$

*with a, b, c, d being non-negative numbers such that c = a + b and d = b + c*.

According to Horadam (1961), the golden ratio ϕ is a natural, feasible fixed point^10^ for the consecutive ratios *b*/*a*, *c*/*b* and *d*/*c* that are related to the generalized 4-length Fibonacci sequence (4). In fact, when *b*/*a* = ϕ, then *c*/*b* = (*a* + *b*)/*b* = 1/ϕ + 1 = ϕ and *d*/*c* = (*b* + *c*)/*c* = 1/ϕ + 1 = ϕ result. In this case, the sequence is described by the model: *y*(*k* + 1) = ϕ*y*(*k*), *k* = 0, 1, 2, where *y*(0) = *a*, *y*(1) = *b*, *y*(2) = *c*, *y*(3) = *d*. One value thus *determines* the whole sequence^11^, in the sense that *the value of just one ratio identically characterizes the whole sequence of ratios* (see the Shannon entropy- based interpretation of Appendix A).

*Non-*{*symmetric and recursive*} *walking*. Define the quantities ΔSW = SW_*l*_ − SW_*r*_, $\Delta {\text{SW}}^{\text{adj}}={\text{SW}}_{l}^{\text{adj}}-{\text{SW}}_{r}^{\text{adj}}$. The following proposition, whose proof again comes from (3), provides the main result of this subsection.

**Proposition 3:***Letc*_*I*_ = ST_*r*_, *c*_*II*_ = ST_*l*_, *d*_*I*_ = GC_*r*_ + ΔSW, *d*_*II*_ = GC_*l*_ − ΔSW*. The two sequences*

(5) $$\begin{array}{l} \;I:\;a_{I},\;b_{I},\;c_{I},\;d_{I}\\ II:\;a_{II},\;b_{II},\;c_{II},\;d_{II} \end{array}$$

*are (generalized) 4-length Fibonacci sequences, generated bya*_*I*_ = DS_*r*_, *b*_*I*_ = SW_*l*_*, and* *a*_*II*_ = DS_*l*_, *b*_*II*_ = SW_*r*_*, respectively. The same holds for the corresponding adjoint sequences*^12^

$$\begin{array}{l} \;I^{\text{adj}}:\;a_{I}^{\text{adj}},\;b_{I}^{\text{adj}},\;c_{I}^{\text{adj}},\;d_{I}^{\text{adj}}\\ {II}^{\text{adj}}:\;a_{II}^{\text{adj}},\;b_{II}^{\text{adj}},\;c_{II}^{\text{adj}},\;d_{II}^{\text{adj}} \end{array}$$

*that involve the quantities:*$a_{I}^{\text{adj}}={\text{DS}}_{r}^{\text{adj}}$, $b_{I}^{\text{adj}}={\text{SW}}_{l}^{\text{adj}}$, $a_{II}^{\text{adj}}={\text{DS}}_{l}^{\text{adj}}$, $b_{II}^{\text{adj}}={\text{SW}}_{r}^{\text{adj}}$, $c_{I}^{\text{adj}}={\text{ST}}_{r}^{\text{adj}}$, $c_{II}^{\text{adj}}={\text{ST}}_{l}^{\text{adj}}$, $d_{I}^{\text{adj}}={\text{GC}}_{r}^{\text{adj}}+\Delta {\text{SW}}^{\text{adj}}$, $d_{II}^{\text{adj}}={\text{GC}}_{l}^{\text{adj}}-\Delta {\text{SW}}^{\text{adj}}$.

Sequences (5) thus constitute multiple—namely, two—copies of (4), with the same happening for the *adjoint* sequences. The golden ratio ϕ here thus possibly occurs as a natural, feasible fixed point for the consecutive ratios *b*_*I*_/*a*_*I*_, *c*_*I*_/*b*_*I*_, *d*_*I*_/*c*_*I*_ and *b*_*II*_/*a*_*II*_, *c*_*II*_/*b*_*II*_, *d*_*II*_/*c*_*II*_, with the same again happening for the *adjoint* sequences.

It is straightforward to note that the mean [element by element] of the two sequences *I* and *II* in (5) is again a (generalized) 4-length Fibonacci sequence of the form (4). Its elements are constituted by the mean double support, mean swing, mean stance, mean gait cycle, respectively [*X*-mean $\bar{X}$ denotes the quantity: (*X*_*l*_ + *X*_*r*_)/2]. The resulting mean sequence again exhibits ϕ as a fixed point for consecutive ratios (apply Proposition 2), so that the same structure of (4) is actually preserved in the non-{symmetric and recursive} case, at the price, however, of just considering the corresponding mean values. All the same happens for the *adjoint* sequences.

A dynamics-on-graph interpretation is provided hereafter. It will lead to a classification of gaits in terms of self-similarity at different magnitudes. The related Shannon-index-based interpretation (Friedkin et al., 2016; Parsegov et al., 2017) can be found in Appendix A.

Let us distinguish again between the symmetric and recursive case and the non-{symmetric and recursive} one.

**Figure 3:**
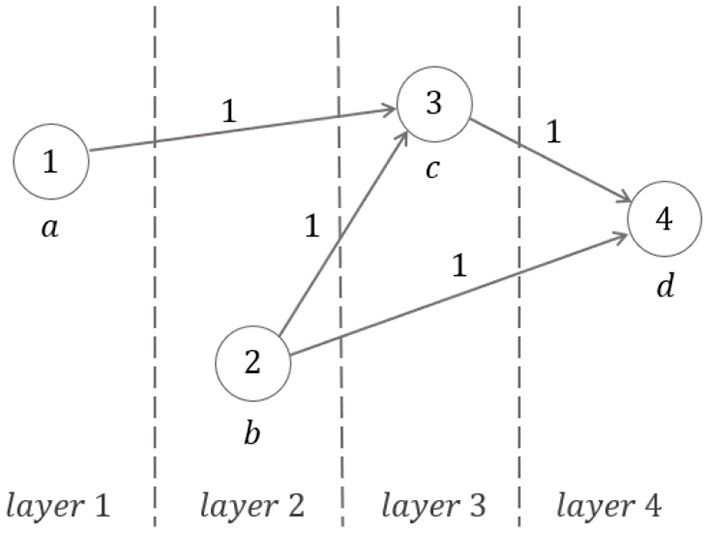
Symmetric and recursive walking: dynamics-on-graph interpretation.

*Symmetric and recursive case*. Consider sequence (4). Let *x*_*j*_ denote the node (or vertex) *j* (belonging to layer *j*) represented in Figure 3 (*j* = 1, 2, 3, 4), with *x*_1_ = *a*, *x*_2_ = *b*, *x*_3_ = *c*, *x*_4_ = *d*.

**Figure 4:**
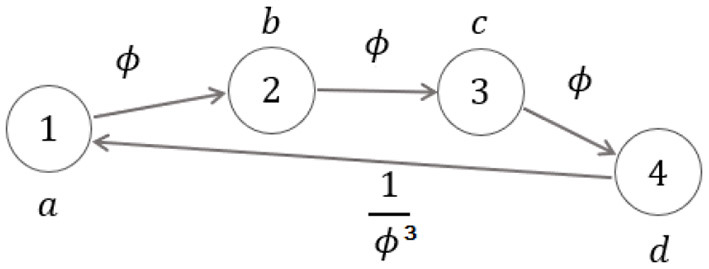
Symmetric and recursive walking with fixed point occurring in the sequence: dynamics-on-graph interpretation.

Write

$$\begin{array}{l} x_{j}=\overset{4}{\underset{i=1}{\sum }}a_{ij}x_{i}+x_{j}\left(0\right), \end{array}$$

where: *x*_*j*_(0) is different from zero only when the input degree of the vertex *j* is equal to zero; *a*_*ij*_ is the (*i, j*)−element of the adjacency-like matrix:

(6) $$\begin{array}{l} A_{d}=\left[\begin{array}{cccc} 0 & 0 & 1 & 0\\ 0 & 0 & 1 & 1\\ 0 & 0 & 0 & 1\\ 0 & 0 & 0 & 0 \end{array}\right], \end{array}$$

showing that the input-degree is either zero or two for any vertex. When the fixed point ϕ occurs for the ratios *b*/*a, c*/*b, d*/*c*, the graph of Figure 3 becomes the strongly connected graph, with all input-degrees being equal to 1 (see Figure 4) and with the corresponding ϕ-dependent adjacency-like matrix reading:

$$\begin{array}{l} A_{d\phi }=\left[\begin{array}{cccc} 0 & \phi & 0 & 0\\ 0 & 0 & \phi & 0\\ 0 & 0 & 0 & \phi \\ \phi^{-3} & 0 & 0 & 0 \end{array}\right]. \end{array}$$

While in the first general case two generating values *determine* the components of the whole graph (in the aforementioned sense), in the second self-similar case just one generating value does it. This is actually the ideal physiological gait of a healthy subject symmetrically and recursively walking at comfortable speed, as described in Iosa et al. (2013).

**Figure 5:**
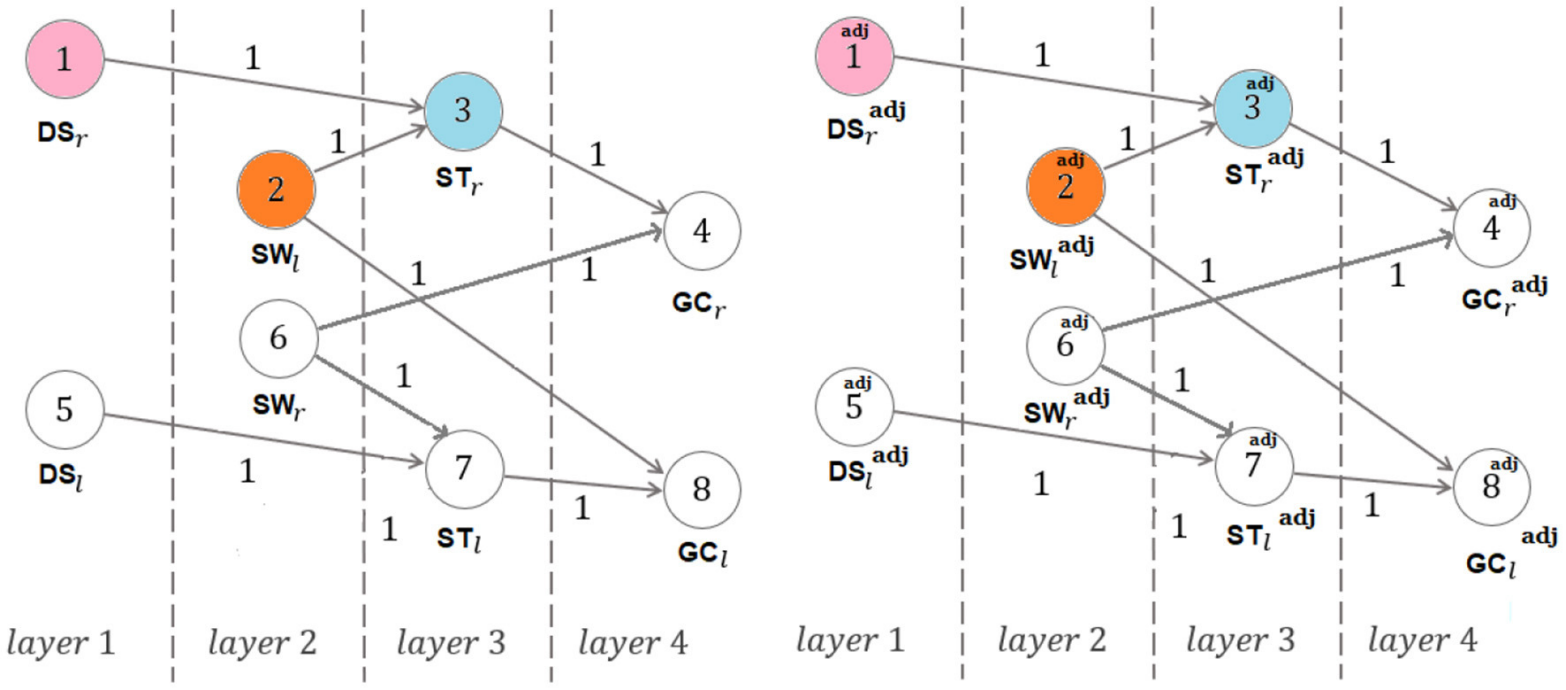
Generic walking: dynamics-on-graph interpretation (same colors denote equal quantities) [${\text{DS}}_{r}^{\text{adj}}={\text{DS}}_{r}$, ${\text{SW}}_{l}^{\text{adj}}={\text{SW}}_{l}$, ${\text{ST}}_{r}^{\text{adj}}={\text{ST}}_{r}$].

*Non-*{*symmetric and recursive*} *case*. Consider sequences (5) and their *adjoint* versions (in their redundant number of four, though the equalities ${\text{DS}}_{r}^{\text{adj}}={\text{DS}}_{r}$, ${\text{SW}}_{l}^{\text{adj}}={\text{SW}}_{l}$, ${\text{ST}}_{r}^{\text{adj}}={\text{ST}}_{r}$ make the two sequences *I* and *I*^adj^ coincident in Proposition 3, according to footnote 12). Let $x_{j}={\left[v_{j},v_{j+4}\right]}^{\text{T}}$ (resp., $x_{j}^{\text{adj}}={\left[v_{j}^{\text{adj}},v_{j+4}^{\text{adj}}\right]}^{\text{T}}$) denote the vector with its components, in order, being constituted by the vertices *v*_*j*_, *v*_*j*+4_ (resp., $v_{j}^{\text{adj}}$, $v_{j+4}^{\text{adj}}$) belonging to layer *j* in Figure 5 (*j* = 1, 2, 3, 4). Write for layers 3 and 4:

$$\begin{array}{lll} x_{j+2} & = & x_{j+1}+\left[\begin{array}{cc} 2-j & j-1\\ j-1 & 2-j \end{array}\right]x_{j},\;j=1,2\\ x_{j+2}^{\text{adj}} & = & x_{j+1}^{\text{adj}}+\left[\begin{array}{cc} 2-j & j-1\\ j-1 & 2-j \end{array}\right]x_{j}^{\text{adj}},\;j=1,2. \end{array}$$

The adjacency-like matrix [characterizing the representation $v_{j}=\overset{8}{\underset{i=1}{\sum }}a_{ij}v_{i}+v_{j}\left(0\right)$ (resp., $v_{j}^{\text{adj}}=\overset{8}{\underset{i=1}{\sum }}a_{ij}v_{i}^{\text{adj}}+v_{j}^{\text{adj}}\left(0\right)$), with *v*_*j*_(0) (resp. $v_{j}^{\text{adj}}\left(0\right)$) being different from zero only when the input degree of the node *j* is equal to zero] for the graph represented in Figure 5, namely:

$$\begin{array}{l} A_{a,d}=\left[\begin{array}{cccccccc} 0 & 0 & 1 & 0 & 0 & 0 & 0 & 0\\ 0 & 0 & 1 & 0 & 0 & 0 & 0 & 1\\ 0 & 0 & 0 & 1 & 0 & 0 & 0 & 0\\ 0 & 0 & 0 & 0 & 0 & 0 & 0 & 0\\ 0 & 0 & 0 & 0 & 0 & 0 & 1 & 0\\ 0 & 0 & 0 & 1 & 0 & 0 & 1 & 0\\ 0 & 0 & 0 & 0 & 0 & 0 & 0 & 1\\ 0 & 0 & 0 & 0 & 0 & 0 & 0 & 0 \end{array}\right], \end{array}$$

can be immediately obtained, again showing that the input-degree is either zero or two for any vertex.

We are now thus able to identify four relevant cases besides the general case in which 6 generating values *determine* the *x*_*j*_-components (resp., $x_{j}^{\text{adj}}$-components) for the whole graph. They are in order (take also into account the interpretation in terms of Shannon entropy of Appendix A):

- 1*-chain adjoint self-similarity* (or *adjoint minimum-entropy in one chain*): just one ratio among *v*_2_/*v*_1_, *v*_6_/*v*_5_, ${v_{6}}^{\text{adj}}/v_{5}^{\text{adj}}$ equals ϕ, with, consequently, just 5 values to *determine* the *x*_*j*_-components for the whole graph.
- 2*-chains adjoint self-similarity* (or *adjoint minimum-entropy in two chains*): two ratios among *v*_2_/*v*_1_, *v*_6_/*v*_5_, ${v_{6}}^{\text{adj}}/v_{5}^{\text{adj}}$ equal ϕ, with, consequently, just 4 values to *determine* the *x*_*j*_-components for the whole graph.
- *Weak adjoint self-similarity* (or *adjoint asymmetric minimum-entropy*): all the three ratios *v*_2_/*v*_1_, *v*_6_/*v*_5_, and ${v_{6}}^{\text{adj}}/v_{5}^{\text{adj}}$ equal ϕ but at least one inequality among *v*_2_ ≠ *v*_6_, $v_{6}\neq v_{6}^{\text{adj}}$, $v_{2}\neq v_{6}^{\text{adj}}$ holds, with, consequently, just 3 or 2 values to *determine* the *x*_*j*_-components for the whole graph.
- *Adjoint symmetric self-similarity* (or *adjoint symmetric minimum-entropy*): all the three ratios *v*_2_/*v*_1_, *v*_6_/*v*_5_, ${v_{6}}^{\text{adj}}/v_{5}^{\text{adj}}$ equal ϕ under the multiple equality $v_{2}=v_{6}=v_{6}^{\text{adj}}$, with, consequently, just 1 value to *determine* the *x*_*j*_-components for the whole graph.
- *Enforced adjoint symmetric self-similarity* (or *enforced adjoint symmetric minimum-entropy*): adjoint symmetric self-similarity in which the double support sub-phases are equally partitioned (*consistency*), that is DS_*x*_ = DS_*y*_ (and DS_*w*_ = Dx_*y*_, DS_*y*_ = DS_*z*_).

**Figure 6:**
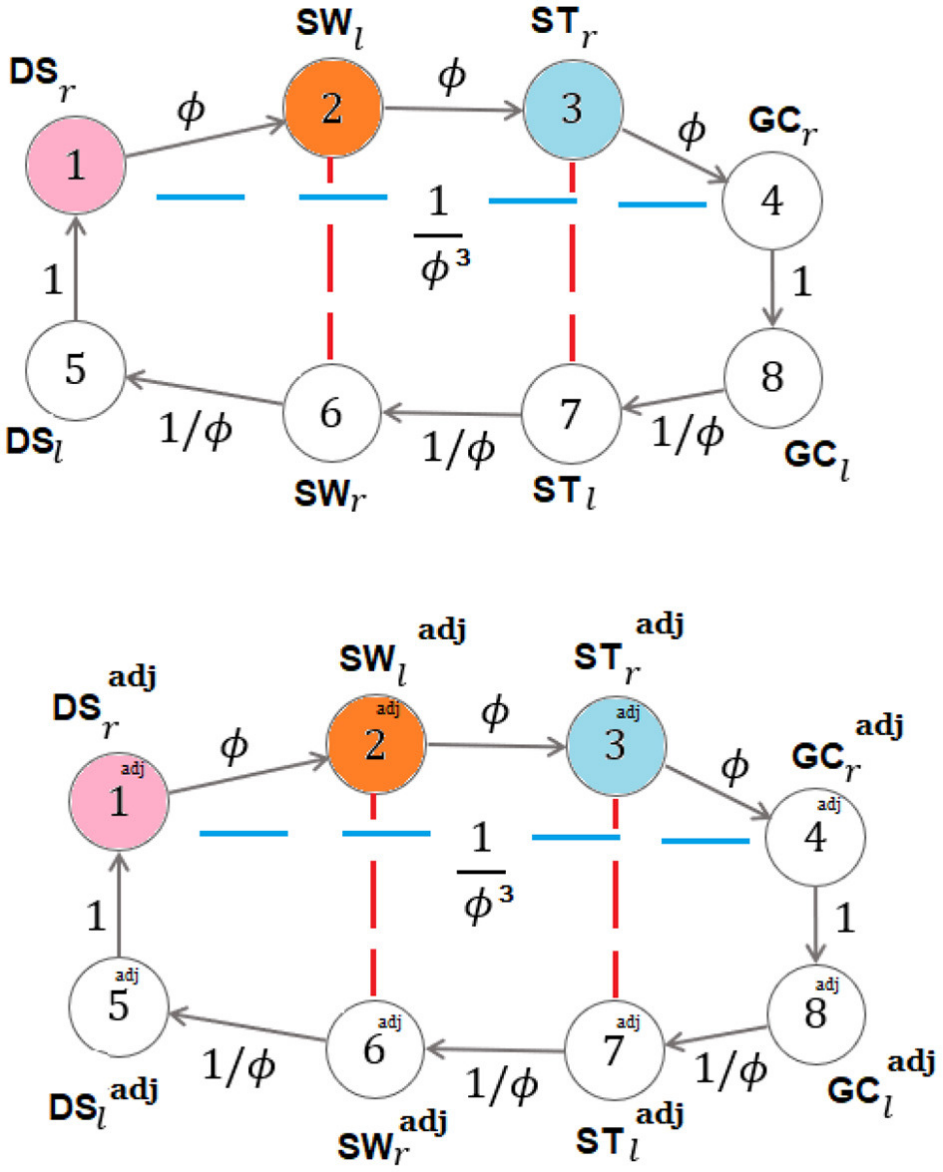
Symmetric and recursive self-similarity for walking with fixed points occurring in all the sequences: dynamics-on-graph interpretation (same colors denote equal quantities) [${\text{DS}}_{r}^{\text{adj}}={\text{DS}}_{r}$, ${\text{SW}}_{l}^{\text{adj}}={\text{SW}}_{l}$, ${\text{ST}}_{r}^{\text{adj}}={\text{ST}}_{r}$].

In the last case, ϕ occurs as a fixed point for the consecutive ratios of the sequences in (4) in a symmetric and recursive setting, so that the graphs of Figure 5 become the (non-minimal-dimension) strongly connected graphs in Figure 6 (in which each graph reproduces the same Figure 4 on its top) with all in degrees being equal to 1 and with the corresponding ϕ-dependent adjacency-like matrix reading:

$$\begin{array}{l} A_{a,d\phi }=\left[\begin{array}{cccccccc} 0 & \phi & 0 & 0 & 0 & 0 & 0 & 0\\ 0 & 0 & \phi & 0 & 0 & 0 & 0 & 0\\ 0 & 0 & 0 & \phi & 0 & 0 & 0 & 0\\ 0 & 0 & 0 & 0 & 0 & 0 & 0 & 1\\ 1 & 0 & 0 & 0 & 0 & 0 & 0 & 0\\ 0 & 0 & 0 & 0 & 1/\phi & 0 & 0 & 0\\ 0 & 0 & 0 & 0 & 0 & 1/\phi & 0 & 0\\ 0 & 0 & 0 & 0 & 0 & 0 & 1/\phi & 0 \end{array}\right]. \end{array}$$

### 2.3. A New Experimental Conjecture

The new experimental conjecture of this subsection extends the ideas underlying a fractal approach to the double support sub-phases within the gait^13^. It is inspired from experimental results reported in Novacheck (1998) showing that physiological symmetric walking is not only characterized by a stance duration being close to 62% of gait cycle duration, a swing duration being close to 38% of gait cycle duration, a double support duration being consequently close to 24% of gait cycle duration, but also by an instant of minimum angular position (with negative sign) of the foot relative to the tibia (with a 90 degrees-angle between foot and tibia being plotted at 0°) occurring at about 7% of gait cycle duration in each double support sub-phase (with 5% as percentage for the complementary interval duration). It may thus be interestingly recognized that the structure of a Fibonacci sequence (with fixed point ϕ) appears in the sequence: 5 × 2 = 10 (1/ϕ^5^ ≈ 9.018); 7 × 2 = 14 (1/ϕ^4^ ≈ 14.591); 24 (1/ϕ^3^ ≈ 23.608); 38 (1/ϕ^2^ ≈ 38.198); 62(1/ϕ ≈ 61.804); 100.

For the sake of clarity, denote by {RHS^[1]^, LTO^[1]^, LHS^[1]^, RTO^[1]^, RHS^[2]^, LTO^[2]^} the sequence of time instants corresponding to the right-heel-strike (FS_*r,a*_, FS_*r,b*_), left-toe-off (FO_*l,a*_, FO_*l,b*_), left-heel-strike (FS_*l,y*_), right-toe-off (FO_*r,y*_) for two subsequent gaits *i* = 1, 2 of Figure 2. The following equalities:

$$\begin{array}{l} \;{\text{DS}}_{r}={\text{LTO}}^{\left[1\right]}-{\text{RHS}}^{\left[1\right]}+{\text{RTO}}^{\left[1\right]}-{\text{LHS}}^{\left[1\right]}\\ \;{\text{DS}}_{l}={\text{RTO}}^{\left[1\right]}-{\text{LHS}}^{\left[1\right]}+{\text{LTO}}^{\left[2\right]}-{\text{RHS}}^{\left[2\right]}\\ {\text{SW}}_{r}={\text{RHS}}^{\left[2\right]}-{\text{RTO}}^{\left[1\right]}\\ {\text{SW}}_{l}={\text{LHS}}^{\left[1\right]}-{\text{LTO}}^{\left[1\right]} \end{array}$$

hold, with ST_*r*_, ST_*l*_, GC_*r*_, GC_*l*_ satisfying (3). We are able to present the following conjecture.

**Conjecture**C**:***Consider the positive real numbersz*_1_, *z*_2_, *z*_3_*denoting the time distances from* RHS^[1]^, LHS^[1]^, RHS^[2]^ *of the three time instants—belonging to the open sets* (RHS^[1]^, LTO^[1]^), (LHS^[1]^, RTO^[1]^), (RHS^[2]^, LTO^[2]^)—*representing the three instants of minimum angular positions (with negative signs) of the (left and right) feet relative to the tibias (with a 90 degrees-angle between foot and tibia being plotted at 0-degrees). The numbers* *z*_1_, *z*_2_, *z*_3_ *are conjectured to characterize the expansion to the left of the (generalized) 4-length Fibonacci sequences (5) into the 6-length (functional) ones [namely*, *b*_I_ = *a*_I_ + (*z*_1_ + *z*_2_) + Δ_I_, *b*_II_ = *a*_II_ + (*z*_2_ + *z*_3_) + Δ_II_ *hold]:*

(7) $$\begin{array}{l} \text{ I}:\;a_{\text{I}}-\left(z_{1}+z_{2}+\Delta_{\text{I}}\left(a_{\text{I}},b_{\text{I}},a_{\text{II}},b_{\text{II}}\right)\right),\;z_{1}+z_{2}\\ \;+\Delta_{\text{I}}\left(a_{\text{I}},b_{\text{I}},a_{\text{II}},b_{\text{II}}\right),\;a_{\text{I}},\;b_{\text{I}},\;c_{\text{I}},\;d_{\text{I}}\\ \text{II}:\;a_{\text{II}}-\left(z_{2}+z_{3}+\Delta_{\text{II}}\left(a_{\text{I}},b_{\text{I}},a_{\text{II}},b_{\text{II}}\right)\right),\;z_{2}+z_{3}\\ \;+\Delta_{\text{II}}\left(a_{\text{I}},b_{\text{I}},a_{\text{II}},b_{\text{II}}\right),\;a_{\text{II}},\;b_{\text{II}},\;c_{\text{II}},\;d_{\text{II}}, \end{array}$$

*with the reals* Δ_I_(·) *and* Δ_II_(·) *being zero (z-specific expansion) when symmetric and self-similar gait occurs (i.e., for* *a*_I_ = *a*_II_ & *b*_I_ = ϕ*a*_I_ = *b*_II_ = ϕ*a*_II_*)*.

The contribution of Δ_I_ and Δ_II_ may be related to energy expenditure, with their zero-nature (*z-specific expansion*) in symmetric and self-similar walking being reminiscent of the physical fact that, at the speed at which symmetric and self-similar walking occurs, locomotor system saves energy and its activity is only required to oppose gravity, to maintain postural configurations and to reintegrate energy loss during each cycle (Mochon and McMahon, 1980; Iosa et al., 2016a). When the conjecture above is verified in symmetric and self-similar walking, the (generalized) 4-length Fibonacci sequences in (5) collapse into one sequence that is being expanded to the left^14^, through *z*_1_, *z*_2_, *z*_3_ only, into the 6-length one:

(8) $$\begin{array}{l} \;a-\left(z_{1}+z_{2}\right),\;z_{1}+z_{2},\;a,\;b,\;c,\;d, \end{array}$$

with each element of the above sequence possessing a clear physical meaning and with ϕ occurring as a fixed point for all the consecutive five ratios: (*z*_1_ + *z*_2_)/(*a* − (*z*_1_ + *z*_2_)), *a*/(*z*_1_ + *z*_2_), *b*/*a*, *c*/*b*, *d*/*c*.

### 2.4. A New Index: The Φ-bonacci Gait Number

Let λ, δ, μ^adj^, λ^adj^, ν^conj^ be positive weights. Given positive reals ξ_*n*_, ξ_*d*_, ξ_*v*_ (where *n* generically stands for numerator, *d* stands for denominator, *v* stands for value), define the normalized quantity^15^:

(9) $$\begin{array}{l} {\left(\frac{\xi_{n}}{\xi_{d}}-\xi_{v}\right)}_{\text{n}}^{2}={\left(\frac{\xi_{n}}{\xi_{d}}\right)}^{-1}{\left(\frac{\xi_{n}}{\xi_{d}}-\xi_{v}\right)}^{2}. \end{array}$$

In accordance with the previously presented classification of gaits, the following index, named Φ*-bonacci gait number*:

(10) $$\begin{array}{l} Y_{\Phi }=\sqrt{{\left(\frac{v_{2}}{v_{1}}-\phi \right)}_{\text{n}}^{2}+{\left(\frac{v_{6}}{v_{5}}-\phi \right)}_{\text{n}}^{2}+\mu^{\text{adj}}{\left(\frac{v_{6}^{\text{adj}}}{v_{5}^{\text{adj}}}-\phi \right)}_{\text{n}}^{2}}\\ \;+\lambda \sqrt{{\left(\frac{v_{6}}{v_{2}}-1\right)}_{\text{n}}^{2}+\lambda^{\text{adj}}{\left(\frac{v_{6}^{\text{adj}}}{v_{6}}-1\right)}_{\text{n}}^{2}}\\ \;+\nu^{\text{conj}}\sqrt{{\left(\frac{a_{I}}{z_{1}+z_{2}}-\phi \right)}_{\text{n}}^{2}+{\left(\frac{a_{II}}{z_{2}+z_{3}}-\phi \right)}_{\text{n}}^{2}}\\ \;+\delta \sqrt{{\left(\frac{{\text{DS}}_{x}}{{\text{DS}}_{y}}-1\right)}_{\text{n}}^{2}} \end{array}$$

is introduced in order to characterize the special case of *enforced adjoint symmetric self-similarity* in walking (recall conjecture C). The expression of such an index in (10), once it is explicitly rewritten as

$$\begin{array}{l} Y_{\Phi }=\sqrt{{\left(\frac{{\text{SW}}_{l}}{{\text{DS}}_{r}}-\phi \right)}_{\text{n}}^{2}+{\left(\frac{{\text{SW}}_{r}}{{\text{DS}}_{l}}-\phi \right)}_{\text{n}}^{2}+\mu^{\text{adj}}{\left(\frac{{\text{SW}}_{r}^{\text{adj}}}{{\text{DS}}_{l}^{\text{adj}}}-\phi \right)}_{\text{n}}^{2}}\\ \;+\lambda \sqrt{{\left(\frac{{\text{SW}}_{r}}{{\text{SW}}_{l}}-1\right)}_{\text{n}}^{2}+\lambda^{\text{adj}}{\left(\frac{{\text{SW}}_{r}^{\text{adj}}}{{\text{SW}}_{r}}-1\right)}_{\text{n}}^{2}}\\ \;+\nu^{\text{conj}}\sqrt{{\left(\frac{{\text{DS}}_{r}}{z_{1}+z_{2}}-\phi \right)}_{\text{n}}^{2}+{\left(\frac{{\text{DS}}_{l}}{z_{2}+z_{3}}-\phi \right)}_{\text{n}}^{2}}\\ \;+\delta \sqrt{{\left(\frac{{\text{DS}}_{x}}{{\text{DS}}_{y}}-1\right)}_{\text{n}}^{2}},\; \end{array}$$

shows that, differently from the area of the *Synchronicity Rectangle* in Marino et al. (2020), (10) takes its minimum zero-value just when *enforced adjoint symmetric self-similarity* under C occurs. It turns out to constitute the most natural generalization, to the non-{symmetric and recursive} walking case, of the corresponding gait ratio |SW/DS−ϕ| defined in Iosa et al. (2013) and Iosa et al. (2016b) for symmetric walking, while it simply incorporates a weighted modification of the index $=|\Delta \text{SW}|/\bar{\text{SW}}$ in Błażkiewicz et al. (2014), evaluated at both the gait and the adjoint gait. A conceptual extension of the use of the Φ*-bonacci gait number* to assess the *gait index variability* along past walking gaits can be naturally introduced, which is briefly reported in Appendix B. A simplified version of the above Φ*-bonacci gait number*, named *s-*Φ*-bonacci gait number* (*s* stands for simplified) can be also derived from the previous expression by setting μ^adj^ = λ^adj^ = ν^conj^ = 0. This leads to

(11) $$\begin{array}{l} Y_{\Phi \left[s\right]}=\sqrt{{\left(\frac{{\text{SW}}_{l}}{{\text{DS}}_{r}}-\phi \right)}_{\text{n}}^{2}+{\left(\frac{{\text{SW}}_{r}}{{\text{DS}}_{l}}-\phi \right)}_{\text{n}}^{2}}\\ \;+\lambda \sqrt{{\left(\frac{{\text{SW}}_{r}}{{\text{SW}}_{l}}-1\right)}_{\text{n}}^{2}}+\delta \sqrt{{\left(\frac{{\text{DS}}_{x}}{{\text{DS}}_{y}}-1\right)}_{\text{n}}^{2}}\; \end{array}$$

that is the weighted sum^16^ of three terms: (i) the first one to account for the self-similarity contribution to the gait generation; (ii) the second one to account for the swing symmetry contribution to the gait generation; (iii) the third one to account for the *double support consistency*, that is the symmetry, within the gait, between the double support sub-phase with the left foot behind and the right foot ahead and the double support sub-phase with the right foot behind and left foot ahead. A zero value for the *s-*Φ*-bonacci gait number* (11) thus describes the case in which self-similarity, swing symmetry, *double support consistency* occur. Such a simplified index turns out to be useful when data concerning the adjoint gaits and the position of the foot relative to the tibia are not available, as in the case of gait analysis reports (like the ones used in section 3.2), just providing mean values of left and right percentages of stance, swing and double support.

### 2.5. Experiments

#### 2.5.1. Experimental Set 1

Experimental results (referred to as results for the *Experimental set 1*) are reported in section 3.1 to test the validity of the conjecture C. *Experimental set 1* is here conceived as a proof of concept. A single healthy subject was tested in two different days—in order to assess phase duration reliability through acquisition *via* different sensor systems (insole, Movit, as described underneath)—and at different speeds—in order to take into account how speed changes are crucial to the presented analysis—. Testing just one healthy subject was considered sufficient at this stage^17^. The main part of the first measurement system is an insole composed of two parts to adapt to different feet and shoes. Four PTF (Polymer Thick Film) force sensors FSR402 (Interlink Electronics Inc, Los Angeles, USA) were placed on the four points characterized by the greatest pressure of the foot (first and second metatarsal head, medial and central heel). The related positions were chosen through the analysis of real walking measurements, acquired by a baropodometric platform and with Pedar® insole (Novel gmch, Germany). They are able to detect the time instants for the first and the last contacts. Secondly, we performed the motion capture and the motion analysis through the Movit System G1 (Captiks, Rome, Italy), which provides accelerometer, gyroscope, magnetometer, quaternion, barometer synced data and is composed of 10-DOF wireless wearable small inertial devices and an USB wireless receiver (Costantini et al., 2018; Ricci et al., 2019a,b; Saggio, 2020).

#### 2.5.2. Experimental Set 2

Experimental results (referred to as results for the *Experimental set 2*) are reported in section 3.2 to illustrate the effectiveness of the *s-*Φ*-bonacci gait number* (11) in explicitly identifying pathological gaits. A secondary analysis was conducted on data collected and published in previous studies (Iosa et al., 2013, 2016a,b). Three groups were selected: (i) group of healthy control subjects (HCS); (ii) group of patients who are characterized by highly asymmetric deficits [such as patients with hemiparetic stroke, (HSP)]; (iii) group of patients who are characterized by an alteration in gait ratio not always being accompanied by motor asymmetries [such as patients with quite symmetric symptoms due to Parkinson's Disease, (PDP)]. The data were extracted from the database according to the following procedure, which was established to accomplish the purposes of the study: (i) extraction of data of subjects in the three groups who are matched per age and per walking speed (this last condition implied the extraction of healthy subjects walking slowly and patients with deficits slightly affecting gait speed); (ii) extraction of data of patients with stroke who are characterized by an evident gait asymmetry and patients with PD who are characterized by deficits slightly impairing their gait asymmetry. These strict criteria allowed us to extract data concerning just 5 subjects within each group.

## 3. Results

### 3.1. Experimental Set 1

Two sets of experiments (3 experiments per set) have been carried out in two different days. The same subject (female, 160 cm, 25 years old, 54 kg) has been involved; 10 meters walking tests in a hallway have been performed at three different speeds for each set of experiments. The temporal analysis concerns two adjacent left and right gaits at steady-state. The sequence of time instants {RHS^[1]^, LTO^[1]^, LHS^[1]^, RTO^[1]^, RHS^[2]^, LTO^[2]^} corresponding to the right-heel-strike, left-toe-off, left-heel-strike, right-toe-off for the two considered subsequent gaits *i* = 1, 2 has been derived from the measurements acquisitions provided by the two (aforementioned) sensor systems. The Movit system has been able even to provide the three time instants *z*_1_, *z*_2_, *z*_3_. All of the results for the *Experimental set 1* are summarized in Tables 1–4. Each of Tables 1–3, concerning experiments at different speeds, report in order (in seconds): (i) the elements of sequence I in (7); (ii) the elements of sequence II in (7); (iii) the consecutive ratios between the elements of sequence I in (7); (iv) the consecutive ratios between the elements of sequence II in (7).

**Table 1:** Experimental data (*Experimental set 1*) concerning the elements of the sequences I and II in (7) for a walking speed equal to 0.97 m/s: first experiment (second experiment).

	***a*_I_−(*z*_1_+*z*_2_)**	***z*_1_+*z*_2_**	***a*_I_ = DS_*r*_**	***b*_I_ = SW_*l*_**	***c*_I_ = ST_*r*_**	***d*_I_ = GC_*r*_+ΔSW**
Movit	0.1018 (0.101)	0.168 (0.169)	0.2698 (0.27)	0.4422 (0.442)	0.712 (0.712)	1.1542 (1.154)
insole			0.269 (0.271)	0.423 (0.44)	0.692 (0.711)	1.115 (1.151)
	***a*_II_−(*z*_2_+*z*_3_)**	***z*_2_+*z*_3_**	***a*_II_ = DS_*l*_**	***b*_II_ = SW_*r*_**	***c*_II_ = ST_*l*_**	***d*_II_ = GC_*l*_−ΔSW**
Movit	0.101 (0.104)	0.169 (0.165)	0.27 (0.269)	0.48 (0.423)	0.75 (0.692)	1.2108 (1.116)
insole			0.265 (0.274)	0.445 (0.426)	0.71 (0.7)	1.152 (1.139)
		$\frac{z_{1}+z_{2}}{{\text{DS}}_{r}-\left(z_{1}+z_{2}\right)}$	**DS_*r*_/(*z*_1_+*z*_2_)**	**SW_*l*_/DS_*r*_**	**ST_*r*_/SW_*l*_**	**(GC_*r*_+ΔSW)/ST_*r*_**
Movit		1.6502 (1.6732)	1.6059 (1.5976)	1.6389 (1.637)	1.6101 (1.6108)	1.6210 (1.6207)
insole				1.5724 (1.6236)	1.6359 (1.6159)	1.6112 (1.6188)
		$\frac{z_{2}+z_{3}}{{\text{DS}}_{l}-\left(z_{2}+z_{3}\right)}$	**DS_*l*_/(*z*_2_+*z*_3_)**	**SW_*r*_/DS_*l*_**	**ST_*l*_/SW_*r*_**	**(GC_*r*_−ΔSW)/ST_*l*_**
Movit		1.6732 (1.5865)	1.5976 (1.6303)	1.777 (1.5724)	1.5625 (1.6359)	1.6144 (1.6127)
insole				1.6792 (1.5547)	1.5955 (1.6431)	1.6225 (1.6271)

**Table 2:** Experimental data (*Experimental set 1*) concerning the elements of the sequences I and II in (7) for a walking speed equal to 1.51 m/s: first experiment (second experiment).

	***a*_I_−(*z*_1_+*z*_2_)**	***z*_1_+*z*_2_**	***a*_I_ = DS_*r*_**	***b*_I_ = SW_*l*_**	***c*_I_ = ST_*r*_**	***d*_I_ = GC_*r*_+ΔSW**
Movit	0.0487 (0.055)	0.0866 (0.099)	0.1353 (0.154)	0.3457 (0.346)	0.481 (0.5)	0.8267 (0.846)
insole			0.147 (0.145)	0.337 (0.349)	0.484 (0.494)	0.821 (0.843)
	***a*_II_−(*z*_2_+*z*_3_)**	***z*_2_+*z*_3_**	***a*_II_ = DS_*l*_**	***b*_II_ = SW_*r*_**	***c*_II_ = ST_*l*_**	***d*_II_ = GC_*l*_−ΔSW**
Movit	0.059 (0.07)	0.0855 (0.104)	0.1445 (0.174)	0.3555 (0.365)	0.5 (0.539)	0.8368 (0.885)
insole			0.149 (0.181)	0.36 (0.36)	0.519 (0.541)	0.873 (0.872)
		$\frac{z_{1}+z_{2}}{{\text{DS}}_{r}-\left(z_{1}+z_{2}\right)}$	**DS_*r*_/(*z*_1_+*z*_2_)**	**SW_*l*_/DS_*r*_**	**ST_*r*_/SW_*l*_**	**(GC_*r*_+ΔSW)/ST_*r*_**
Movit		1.7782 (1.8)	1.5623 (1.5555)	2.555 (2.2467)	1.3913 (1.445)	1.7187 (1.692)
insole				2.2925 (2.4068)	1.4362 (1.4154)	1.6962 (1.7064)
		$\frac{z_{2}+z_{3}}{{\text{DS}}_{l}-\left(z_{2}+z_{3}\right)}$	**DS_*l*_/(*z*_2_+*z*_3_)**	**SW_*r*_/DS_*l*_**	**ST_*l*_/SW_*r*_**	**(GC_*r*_−ΔSW)/ST_*l*_**
Movit		1.4491 (1.4857)	1.69 (1.673)	2.4602 (2.0977)	1.4064 (1.4767)	1.6736 (1.6419)
insole				2.4161 (1.9889)	1.4416 (1.5027)	1.6820 (1.6118)

**Table 3:** Experimental data (*Experimental set 1*) concerning the elements of the sequences I and II in (7) for a walking speed equal to 0.85 m/s (walking speed equal to 1.1 m/s).

	***a*_I_−(*z*_1_+*z*_2_)**	***z*_1_+*z*_2_**	***a*_I_ = DS_*r*_**	***b*_I_ = SW_*l*_**	***c*_I_ = ST_*r*_**	***d*_I_ = GC_*r*_+ΔSW**
Movit	0.104 (0.0932)	0.145 (0.156)	0.249 (0.2492)	0.462 (0.4428)	0.7115 (0.692)	1.173 (1.1348)
Insole			0.243 (0.23)	0.467 (0.43)	0.71 (0.66)	1.177 (1.09)
	***a*_II_−(*z*_2_+*z*_3_)**	***z*_2_+*z*_3_**	***a*_II_ = DS_*l*_**	***b*_II_ = SW_*r*_**	***c*_II_ = ST_*l*_**	***d*_II_ = GC_*l*_−ΔSW**
Movit	0.09 (0.0939)	0.141 (0.1562)	0.231 (0.2501)	0.442 (0.423)	0.673 (0.6731)	1.114 (1.0954)
Insole			0.223 (0.23)	0.44 (0.41)	0.663 (0.64)	1.06 (1.05)
		$\frac{z_{1}+z_{2}}{{\text{DS}}_{r}-\left(z_{1}+z_{2}\right)}$	**DS_*r*_/(*z*_1_+*z*_2_)**	**SW_*l*_/DS_*r*_**	**ST_*r*_/SW_*l*_**	**(GC_*r*_+ΔSW)/ST_*r*_**
Movit		1.3942 (1.6738)	1.7172 (1.5974)	1.8554 (1.7768)	1.54 (1.5627)	1.6486 (1.6398)
Insole				1.9218 (1.8695)	1.5203 (1.5348)	1.6577 (1.6515)
		$\frac{z_{2}+z_{3}}{{\text{DS}}_{l}-\left(z_{2}+z_{3}\right)}$	**DS_*l*_/(*z*_2_+*z*_3_)**	**SW_*r*_/DS_*l*_**	**ST_*l*_/SW_*r*_**	**(GC_*r*_−ΔSW)/ST_*l*_**
Movit		1.5666 (1.6634)	1.6382 (1.6011)	1.9134 (1.6913)	1.5226 (1.5912)	1.6552 (1.6273)
Insole				1.9730 (1.7826)	1.5068 (1.5609)	1.5987 (1.6406)

**Table 4:** Consistency indices (*Experimental set 1*).

**Walking speed v (m/s)**	**Mean value Δ(*v*)**	***M*_ϕ_(*v*)**	***M*_ϕ,*e*_(*v*)**
0.97	0.01485	0.0567	0.0567
1.1	0.02715	0.1588	0.1588
0.85	0.069	0.2954	0.2954
1.51	0.107325	0.78285	0.78285

*Showing data consistency from different sensor systems*. The Bland-Altman analysis, which corresponds to the 24 couples of independently measured values *a*_*i*_, *b*_*i*_ (*i* = I,II) in Tables 1–3 coming from Movit and insole, respectively, shows: bias = 0.005046; standard deviation of bias = 0.01083; 95% limits of agreement = {−0.01619, 0.02628}, whereas the related two sequences (Movit and insole, respectively) exhibit: mean = {0.3181, 0.3131}; standard deviation = {0.10996, 0.10674}; lower 95% CI of mean = {0.2717, 0.2680}; upper 95% CI of mean = {0.3646, 0.3582}.

*Showing data consistency from repeated experiments*. The Bland-Altman analysis, which corresponds to the 12 couples of measured values (*z*_1_ + *z*_2_), (*z*_2_ + *z*_3_), *a*_*i*_, *b*_*i*_ (*i* = I,II) in Tables 1, 2 for the first and the second experiment (mean = 0.2460, 0.2463), respectively, shows: bias = −0.002325; standard deviation of bias = 0.02134; 95% limits of agreement = {−0.04415, 0.0395}.

*Showing occurrence of Fibonacci sequences*. The sequences in (5): *a*_*i*_, *b*_*i*_, *c*_*i*_, *d*_*i*_ (*i* = I,II) of Proposition 3 are confirmed to exactly constitute generalized 4-length Fibonacci sequences for each set of the specific sensor system acquisitions (see again Tables 1–3), with all the ratios between consecutive elements of such sequences being pretty close to the golden ratio ϕ (especially look at the last ratio) just when the walking speed is 0.97 m/s (approximately constituting the comfortable walking speed for the 160 cm/54 kg- subject under investigation) and relatively different from ϕ as the walking speed differs from 0.97 m/s (see the bottom-halves of Tables 1–3).

*Showing consistency of Conjecture*C. The smaller the difference between the walking speed and 0.97 m/s is, the more the sequences (7) are close to constitute the (generalized) 6-length Fibonacci sequence (8), with all the ratios between consecutive elements of such sequences being close to the golden ratio ϕ. This can be seen by considering the following consistency indices reported in Table 4: mean values Δ(*v*) of all |Δ_I_| and |Δ_II_| [from the two experiments when available] at different speeds *v* (Tables 1–3); maximum modulus of the mean distance *M*_ϕ_(*v*) from ϕ [mean from first and second experiment] of all the ratios [Movit] between consecutive elements in sequences (5) at different speeds *v* (Tables 1–3); maximum modulus of the mean distance *M*_ϕ,*e*_(*v*) from ϕ [mean from first and second experiment] of all the ratios [Movit] between consecutive elements in sequences (7) at different speeds *v* (Tables 1–3).

### 3.2. Experimental Set 2

Even though severe gait deficits may lead to significant differences in most of the spatio-temporal gait parameters (w.r.t. HCS), 15 subjects (5 HCS, 5 HSP, 5 PDP) were selected, who are not only age-matched but also walking-speed-matched. Starting from the values of the gait phases, the (left, right) Gait Ratio GR was computed as the percentage ratio between the (left, right) gait cycle duration and (left, right) stance duration, while the Mean Gait Ratio MGR was given by the average between the left and right GRs. Then, the Symmetry Index SI was computed as the highest GR divided by the smallest GR (among the two feet). The *s-*Φ*-bonacci gait number* $Y_{\Phi \left[s\right]}$ (11) was finally computed (λ = δ = 1) and used for comparison. All of the results for the *Experimental set 2* are summarized in Table 5. In particular, Table 5 shows that, despite the similar speeds, the MGR resulted significantly different among the three groups: differences were observed in PDP (*p* = 0.008 vs. HCS, *post-hoc* analysis), whereas no relevant differences were observed in HSP (*p* = 0.754 vs. HCS). However, as expected, the symmetry between the gait ratio evaluated between the left and the right feet resulted lost in HSP (*p* = 0.009 vs. HCS), more than in PDP (*p* = 0.222 vs. HCS). These results confirm that our extraction was effective in finding two groups of slightly severely affected patients, one most in gait harmony (PDP, as also reported in Iosa et al., 2016b), and the other one in gait symmetry (HSP, as also reported in Iosa et al., 2016a).

**Table 5:** (*Experimental set 2*): Mean ± standard deviations for age and spatio-temporal gait parameters, computed for patients with Parkinson's Disease (PDP), patients with hemiparetic stroke (HSP) and healthy control subjects (HCS) and compared by Kruskal-Wallis analysis, whose *p*-values are reported in the last column [in bold if statistically significant, whereas the symbol * (besides bold characters) highlights statistical significant differences with respect to HCS at *post-hoc* analyses].

	**PDP**	**HSP**	**HCS**	**Kruskal-Wallis analysis**
Age (years)	67.6 ± 6.7	64.2 ± 2.0	63.2 ± 2.2	0.288
Walking speed (m/s)	0.99 ±0.28	0.88 ± 0.19	1.02 ± 0.11	0.980
MGR	**1.51** ± 0.07*	1.62 ± 0.06	1.62 ± 0.02	**0.018**
SI	1.02 ± 0.02	**1.11** ± 0.07*	1.01 ± 0.01	**0.008**
$Y_{\Phi \left[s\right]}$	**1.09** ± 0.38*	**0.95** ± 0.51*	0.21 ± 0.10	**0.011**

## 4. Discussion

Previous results showed that the MGR is close to golden ratio for healthy subjects (Iosa et al., 2013), and far from it for PDP (Iosa et al., 2016b). For patients with stroke, it has been shown that the MGR is strictly related to speed (Iosa et al., 2016a), and hence the MGR of a group walking at a speed that is not significantly lower than the healthy subjects' one, was not expected to be significantly different from the MGR of a healthy subject, as our data here explicitly illustrate. On the other hand, patients with stroke, owing to their hemiparesis, exhibited a more asymmetric gait than HCS and PDP. According to Table 5, the *s-*Φ*-bonacci gait number* $Y_{\Phi \left[s\right]}$ (11) was simultaneously able to find statistically significant differences between PDP and HCS (*p* = 0.008), and also between HSP and HCS (*p* = 0.016). Furthermore, the compensation strategies adopted by the non-paretic limb made the MGR in HSP similar to the healthy subjects' one, but achieved through an asymmetric and less reliable walking. In other words, the *s-*Φ*-bonacci gait number* $Y_{\Phi \left[s\right]}$ effectively merged the asymmetry with the role of the proportions among gait phases, resulting statistically significant for both the disharmonic gait of PDP and the asymmetric gait of HSP.

Indeed, the main advantage of the Φ*-bonacci gait number* (10) [even in its simplified version (11)] is to consider, in a comprehensive manner, symmetry and harmony of walking in terms of gait phases. Such gait phases have been analyzed since the birth of gait analysis, thought as a science to analyse human movement in a quantitative manner (Perry, 1992). Changes in stance, swing and double support phases are strictly related and intertwined between the two feet (Perry, 1992), so that it turns out to be important assessing such changes in an unique meaningful index. Indeed, when compared to the three gait ratios already proposed in previous studies (Iosa et al., 2016b; Serrao et al., 2017), (10) and (11) have the advantage to consider also asymmetry between right and left lower limb kinematics. These indices are theoretically close to 0 for a perfectly harmonic and symmetric gait and far from 0 for a pathological gait. On the other hand, the analysis performed in the *Experimental set 1* has preliminarily shown consistency of data at the root of such an index computation and phase duration reliability through acquisition *via* different sensor systems. The concurrent validity of the new index [in its version (11)] has been illustrated through the *Experimental set 2*, highlighting the differences between healthy subjects, subjects with hemiparetic stroke characterized by an asymmetric walking, and patients with Parkinson's Disease characterized by a non-harmonic walking.

Anyway, the results of the present study should be interpreted with caution, owing to its specific limits, such as the small size of the samples enrolled in the two experimental sets. Many aspects should be further investigated in future studies. One of them relies on the fact that the Φ*-bonacci gait number* is focused on the proportions among the gait phases, limiting this type of gait analysis to temporal features. It could be certainly interesting to put it in relationship with [and test the correlation between (10)–(11) and quantitative indices related to] the role played by different sensory information in maintaining the gait harmony^18^. On the other hand, an additional potential limit of the present study is that we compared the *s-*Φ*-bonacci gait number* in healthy subjects and patients with stroke or Parkinson's Disease, but if it has to be useful in clinical settings, then this index should exhibit responsiveness to small changes obtained with rehabilitation.

In spite of such aforementioned limits, the present study also possesses points of strength, since it is based on a wide literature illustrating how gait phases are a reliable and valid measure of subject's walking. Even though the capability of the Φ*-bonacci gait number* in highlighting within-subject changes in walking has not been explicitly tested, previous studies have already shown that rehabilitative interventions were able to modify the phases of gait cycle in patients with Parkinson's Disease. In light of the reported changes in terms of stance, swing and double support phases, we might reasonably suppose that also the Φ*-bonacci gait number* can detect patients improvements due to rehabilitation, especially in terms of self-similarity, symmetry, consistency of the gaits as a valid and repetitive measure for the assessment of walking ability (Teufl et al., 2018).

## 5. Conclusions

Healthy and pathological human walking have been characterized from a temporal point of view in terms of two sets of eight specific time intervals concerning the *composite gait cycle*. The corresponding mathematical description in terms of generalized finite-length Fibonacci sequences and dynamics-on-graph concepts has naturally explained the crucial role of the golden ratio ϕ, while extending the related analyses in Iosa et al. (2013) and Marino et al. (2020). An interpretation in terms of Shannon entropy is also included in Appendix A. The new gait index (10), named Φ*-bonacci gait number* has been defined to assess recursivity, asymmetry, consistency, and self-similarity of the gait. It relies on a new experimental conjecture that concerns an extended fractal walking decomposition paying attention on the position of the foot relative to the tibia. Experimental results concerning the simplified version (11) of the index (10) have supported the theoretical derivations. Besides the aforementioned contributions, this paper may even provide new perspectives for developing quantitative assessment of human walking, efficient humanoid robotic walkers, and effective neuro-robots for rehabilitation, in line with the related discussion in the recent (Iosa et al., 2017). Finally, by repeatedly extending the application of the adjoint gait (i.e., adjoint gait of the adjoint gait and so on), a collection of indices representing overlapping gaits can be constructed and gait index variability along past walking gaits can be accordingly assessed in a natural way, as described in Appendix B.

## Data Availability Statement

The raw data supporting the conclusions of this article will be made available by the authors, without undue reservation.

## Ethics Statement

Ethical review and approval was not required for the study on human participants in accordance with the local legislation and institutional requirements. Written informed consent for participation was not required for this study in accordance with the national legislation and the institutional requirements.

## Author Contributions

CV and MI: conceptualization, writing the review, and editing. CV, MI, PR, and GS: methodology. MI and GS: software and resources. CV, MI, AP, FG, and GS: validation and investigation. CV, MI, and FG: formal analysis. CV: writing the original draft. All authors contributed to the article and approved the submitted version.

## Conflict of Interest

The reviewer AR declared an affiliation with INAIL, which provided partial support for this work within the framework of the BRIC project: Project STAR - Innovative STrategies, and Approaches for the motor and functional Rehabilitation of subjects with neurovascular adverse event outcomes for reintegration into work. The reviewer MT declared a past co-authorship with one of the authors MI to the handling Editor.

## Publisher's Note

All claims expressed in this article are solely those of the authors and do not necessarily represent those of their affiliated organizations, or those of the publisher, the editors and the reviewers. Any product that may be evaluated in this article, or claim that may be made by its manufacturer, is not guaranteed or endorsed by the publisher.

## Figures and Tables

**Figure 7:**
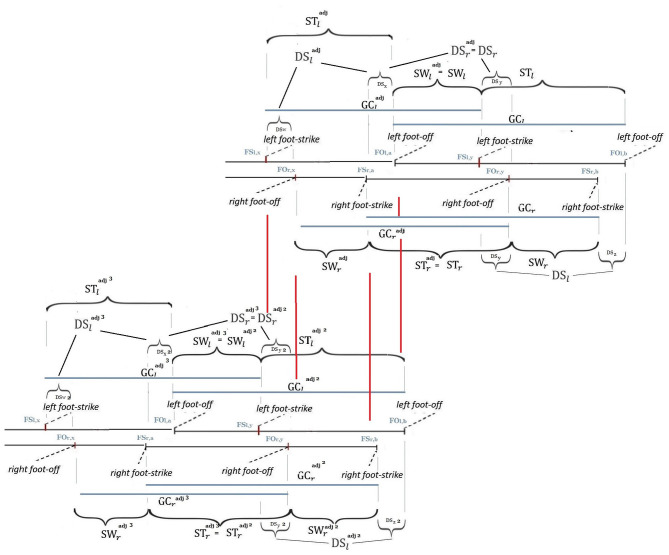
Walking gaits and their repetitive adjoints.

## Footnotes

^1^Spatio-temporal gait parameters are analyzed in Iosa et al. (2013) by using a stereo-photogrammetric system with 6 cameras.

^2^It is worth noting that a similar percentage 61.06% ≡ 1 − ϕ = 1/ϕ is the same that appears, in a normal subject performing a head-up tilt, as the ratio between the LF (low frequency) component and the total power in the Heart Rate Variability context (The European Society of Cardiology & the North American Society of Pacing & Electrophysiology, 1996, pag. 361, Figure 5.f), whereas 38.94% ≡ 1/ϕ^2^ equals the ratio between the LF total-power-complement and the total power.

^3^The generic idea that walking can have a fractal structure can be found in Hausdorff et al. (1995), while the existence of significant alterations from such a structure in patients with Parkinson's Disease has been suggested in Hausdorff et al. (2003).

^4^As reported in Igamberdiev (2004), certain recursive limits become fundamental canons of perfection formed as memorization within reflective loops.

^5^With respect to this, notice that, even in the symmetric and recursive case, the ratio between swing and double support phases durations in healthy subjects walking at comfortable speed slightly differs from ϕ, so that a perfectly self-similar gait does not occur in practice, with a consequent mismatch arising between occurred temporal events and currently available related analysis.

^6^A completely different use of Fibonacci numbers is, instead, suggested in Iosa et al. (2017), which is related to the interpretation of ϕ as the convergent equilibrium of the Ultimatum Game.

^7^The swing duration is sometimes denoted by SS, with SS standing for Single Support.

^8^For the sake of clarity, we do not explicitly use, in this subsection, the fact that ${\text{DS}}_{r}^{\text{adj}}={\text{DS}}_{r}$, ${\text{SW}}_{l}^{\text{adj}}={\text{SW}}_{l}$, ${\text{ST}}_{r}^{\text{adj}}={\text{ST}}_{r}$.

^9^Note that, in the case of DS = 0 (namely, in the limit case at which walking switches into running), sequence (4)—normalized with respect to SW—, reduces to the classical Fibonacci sequence generated by 0, 1.

^10^The other fixed point (1 − ϕ) for the consecutive ratios *b*/*a*, *c*/*b* and *d*/*c* is not feasible when, as in this case, *a* and *b* are non-negative.

^11^Notice the meaningful similarity between the 4-length sequence *a* = DS, *b* = SW, *c* = ST, *d* = GC (in the walking cycle) and the 4-length sequence *a* = D-S-2IV_*r*_, *b* = S+IV_*r*_, *c* = I_*f*_, *d* = R-R (in the cardiac cycle measured after 15 min of rest at supine position), both of them with ϕ as fixed point for the three consecutive ratios *d*/*c*, *c*/*b*, *b*/*a* (Ozturk et al., 2016), where IV_*r*_ is the duration of the iso-volumic relaxation phase, D is the diastole duration, S is the systole duration, I_*f*_ is the inflow duration, R-R is the interval between two consecutive heart beats. Even notice correspondences with the latest analysis of the ratios of phase durations in the front crawl swimming stroke in Verrelli et al. (2021).

^12^Notice that the two sequences *I* and *I*^adj^ coincide, owing to the equalities: ${\text{DS}}_{r}^{\text{adj}}={\text{DS}}_{r}$, ${\text{SW}}_{l}^{\text{adj}}={\text{SW}}_{l}$, ${\text{ST}}_{r}^{\text{adj}}={\text{ST}}_{r}$.

^13^The *adjoint gait* is not considered in this subsection, since recursivity has been already addressed in the preceding subsection.

^14^Note that extending such an argument up to the case of infinite-length Fibonacci sequences would lead to asymptotic self-similarity generation. In fact, the limit behavior:

$$\begin{array}{l} \underset{k\to +\infty }{\lim}\frac{y_{i}\left(k+1\right)}{y_{i}\left(k\right)}=\underset{k\to +\infty }{\lim}\frac{\phi^{k+1}\beta_{i}-{\left(1-\phi \right)}^{k}\alpha_{i}}{\phi^{k}\beta_{i}+{\left(1-\phi \right)}^{k}\phi \alpha_{i}}\;=\;\phi \end{array}$$

holds.

^15^Different normalizations can be apparently used.

^16^The gains within can be freely chosen by the user, in accordance with the specific analysis requirements.

^17^As we shall see, a larger number of subjects will be enrolled for the *Experimental set 2*.

^18^For instance, further studies should clarify the role played by vision (owing to the fact that a reduction of upper body stability and harmony has been exhibited by subjects walking blindfolded Iosa et al., 2012) or by the vestibular system that could be involved in maintaining balance during locomotion (Bent et al., 2005).

## Appendix A

### Shannon-Index-Based Interpretation

Consider the generalized Fibonacci sequence: *a*, *b*, *c*, *d* (generally arising from the symmetric & recursive or the non-{symmetric & recursive} cases of Propositions 2 and 3, respectively), along with the three ratios: *b*/*a*, *c*/*b*, *d*/*c*. Look for a suitably defined string of characters and a corresponding Shannon-index characterization of the case in which the golden ratio ϕ is a fixed point for the consecutive ratios *b*/*a*, *c*/*b* and *d*/*c* and one value determines the whole sequence. With respect, recall that the Shannon index (or diversity index) may be used to quantify the entropy in a string of text (Spellerberg and Fedor, 2003). The more different letters there are, the more difficult it is to correctly predict which letter will be the next one in the string. To this purpose, take the three differences *d*_1_ = *b*/*a* − *c*/*b*, *d*_2_ = *d*/*c* − *c*/*b*, *d*_3_ = *b*/*a* − *d*/*c* and let *M*_*R*_ be a sufficiently large positive odd integer. Let P be a finite partition of the compact set [−*M*_*R*_, *M*_*R*_], with disjoint blocks (or cells) $A_{j}$ of the form:

$$\begin{array}{l} A_{j}=\left[x_{j},x_{j+1}\right],\;j=1,2,\ldots ,M_{R}-1,\\ A_{j}=\left[x_{j},x_{j+1}\right],\;j=M_{R}, \end{array}$$

where *x*_*j*+1_ = *x*_*j*_ + 2, *j* = 1, 2, …, *M*_*R*_, and *x*_1_ = −*M*_*R*_. Let $P_{l}$ a finite refinement of P (*l* = 0, 1, …, *R*_*l*_, *R*_*l*_ is a sufficiently large natural number), with finer blocks $A_{k\left[j\right]}^{\left[l\right]}\subset A_{j}$ of the form:

$$\begin{array}{l} A_{k\left[j\right]}^{\left[l\right]}=\left[x_{k\left[j\right]}^{\left[l\right]},x_{k+1\left[j\right]}^{\left[l\right]}\right), \end{array}$$

where $x_{k+1\left[j\right]}^{\left[l\right]}=x_{k\left[j\right]}^{\left[l\right]}+1/2^{l}$, *k* = 1, …, 2^(*l*+1)^, and $x_{1\left[j\right]}^{\left[l\right]}=x_{j}$. For each *l* = 0, 1, …, *R*_*l*_, define the set of characters (or letters)

$$\begin{array}{l} \Sigma^{\left[l\right]}=\left\{x_{1}=x_{1\left[1\right]}^{\left[l\right]},\ldots ,x_{2^{\left(l+1\right)}+1\left[1\right]}^{\left[l\right]}=x_{2}=x_{1\left[2\right]}^{\left[l\right]},\ldots ,\ldots \right\}. \end{array}$$

Consider the string of characters: $\left(s_{1}^{\left[l\right]},s_{2}^{\left[l\right]},s_{3}^{\left[l\right]}\right)$, where $s_{1}^{\left[l\right]}$, $s_{2}^{\left[l\right]}$, $s_{3}^{\left[l\right]}$ belong to the above set Σ^[*l*]^ and $s_{m}^{\left[l\right]}$ (*m* = 1, 2, 3) equals the smallest element of $A_{k\left[j\right]}^{\left[l\right]}$ when the difference *d*_*m*_ belongs to the block $A_{k\left[j\right]}^{\left[l\right]}$. Let $p_{*r}^{\left[l\right]}$ be the number of characters belonging to the *r*-th character type in the three-elements-string $\left(s_{1}^{\left[l\right]},s_{2}^{\left[l\right]},s_{3}^{\left[l\right]}\right)$ divided by 3 (*r* = 1, …, *N*^[*l*]^, *N*^[*l*]^ ≤ 3). Finally take the Shannon index for such a string $\left(s_{1}^{\left[l\right]},s_{2}^{\left[l\right]},s_{3}^{\left[l\right]}\right)$ as

$$\begin{array}{l} H_{s}^{\left[l\right]}=-\overset{N^{\left[l\right]}}{\underset{r=1}{\sum }}p_{*r}^{\left[l\right]}\text{ln}\left(p_{*r}^{\left[l\right]}\right), \end{array}$$

where $\overset{N^{\left[l\right]}}{\underset{r=1}{\sum }}p_{*r}^{\left[l\right]}=1$. The more unequal the abundances of types in the string is, the smaller the corresponding Shannon entropy is made, with $H_{s}^{\left[l\right]}$ satisfying^19^

$$\begin{array}{l} H_{s}^{\left[l\right]}\in \left[0,\text{ln }\left(N^{\left[l\right]}\right)\right]. \end{array}$$

In particular, if all abundance is concentrated to one type, Shannon entropy is zero and there is no uncertainty in predicting the type of the next entity. The case in which the golden ratio ϕ is a fixed point for the consecutive ratios *b*/*a*, *c*/*b* and *d*/*c* is thus characterized by the condition $\overset{R_{l}}{\underset{l=1}{\sum }}H_{s}^{\left[l\right]}=0$, for any *R*_*l*_ ∈ ℕ ∪ {0}.

## Appendix B

Introduce the definition of *adjoint* application, i.e.: *given any left or right gait cycle defined as the time interval between two consecutive lift off or two consecutive strikes of the corresponding foot, its adjoint is defined as the time interval between two consecutive strikes or two consecutive foot off of the corresponding foot, respectively, starting from the strike or the lift off immediately preceding the lift off or the strike of the considered gait, respectively*. Denote the adjoint of the adjoint gait by the symbol adj2, whereas denote the adjoint of the adjoint of the adjoint gait by the symbol adj3 (see Figure 7). The phases within such gaits inherit the same notation. Figure 7 shows that, by definition, ${\text{SW}}_{r}^{\text{adj}2}={\text{SW}}_{r}^{\text{adj}}$, ${\text{ST}}_{l}^{\text{adj}2}={\text{ST}}_{l}^{\text{adj}}$, ${\text{DS}}_{l}^{\text{adj}2}={\text{DS}}_{l}^{\text{adj}}$, whereas DS_*y*2_ and DS_*z*2_ [that characterize the double support ${\text{DS}}_{l}^{\text{adj}2}$] satisfy the seam-constraints: DS_*y*2_ = DS_*w*_, DS_*z*2_ = DS_*x*_. The Φ*-bonacci gait number* at stage −1 can be thus defined as^20^

(12) $$\begin{array}{l} Y_{\Phi ,-1}=[{\left(\frac{{\text{SW}}_{l}^{\text{adj2}}}{{\text{DS}}_{r}^{\text{adj2}}}-\phi \right)}_{\text{n}}^{2}+{\left(\frac{{\text{SW}}_{r}^{\text{adj2}}}{{\text{DS}}_{l}^{\text{adj2}}}-\phi \right)}_{\text{n}}^{2}\\ {\;+\mu_{-1}^{\text{adj}}{\left(\frac{{\text{SW}}_{r}^{\text{adj3}}}{{\text{DS}}_{l}^{\text{adj3}}}-\phi \right)}_{\text{n}}^{2}]}^{1/2}+\lambda_{-1}[{\left(\frac{{\text{SW}}_{r}^{\text{adj2}}}{{\text{SW}}_{l}^{\text{adj2}}}-1\right)}_{\text{n}}^{2}\\ {\;+\lambda_{-1}^{\text{adj}}{\left(\frac{{\text{SW}}_{r}^{\text{adj3}}}{{\text{SW}}_{r}^{\text{adj2}}}-1\right)}_{\text{n}}^{2}]}^{1/2}+\nu_{-1}^{\text{conj}}[{\left(\frac{{\text{DS}}_{r}^{\text{adj2}}}{z_{1}^{\text{adj2}}+z_{2}^{\text{adj2}}}-\phi \right)}_{\text{n}}^{2}\\ {\;+{\left(\frac{{\text{DS}}_{l}^{\text{adj2}}}{z_{2}^{\text{adj2}}+z_{3}^{\text{adj2}}}-\phi \right)}_{\text{n}}^{2}]}^{1/2}+\delta_{-1}{\left[{\left(\frac{{\text{DS}}_{x2}}{{\text{DS}}_{y2}}-1\right)}_{\text{n}}^{2}\right]}^{1/2}, \end{array}$$

where $\mu_{-1}^{\text{adj}}$, λ_−1_, $\lambda_{-1}^{\text{adj}}$, $\nu_{-1}^{\text{conj}}$, δ_−1_ are positive gains that play the same role of μ^adj^, λ, λ^adj^, ν^conj^, δ, while ${\text{DS}}_{x2}+{\text{DS}}_{y2}={\text{DS}}_{r}^{\text{adj}2}$. Gait index variability can be naturally assessed by comparing the Φ*-bonacci gait numbers* at different stages, through the diversity index computed on the basis of their approximated values (in accordance with certain required tolerances). Notice that if the ordered-in-time sequence of left and right foot strike and off is translated into a sequence of beats, then the same tools used to quantify the Heart Rate Variability can be adopted (Hausdorff, 2005). In this respect, a perfectly self-similar, symmetric, consistent, recursive, gait-index-stable walking exhibits a power spectral density with exclusive content at the frequency 2/GC (in Hz if GC measured in seconds). Evaluating gait fluctuations can be used as a complementary way of quantifying gait reliability with respect to age and disease, as well as a means of monitoring the effects of therapeutic interventions and rehabilitation (Hausdorff, 2005), provided that this approach is actually based on all the foot strike and the foot off events.
